# Spatial transferability of an agent-based model to simulate *Taenia solium* control interventions

**DOI:** 10.1186/s13071-023-06003-9

**Published:** 2023-11-08

**Authors:** Francesco Pizzitutti, Gabrielle Bonnet, Eloy Gonzales-Gustavson, Sarah Gabriël, William K. Pan, Armando E. Gonzalez, Hector H. Garcia, Seth E. O’Neal, for the Cysticercosis Working Group in Peru

**Affiliations:** 1 Geography Department, San Francisco de Quito University, Quito, Ecuador; 2 Centre for Mathematical Modelling of Infectious Disease (CMMID), Department of Infectious Disease Epidemiology, London School of Hygiene & Tropical Medicine, London, UK; 3 Tropical and Highlands Veterinary Research Institute, Universidad Nacional Mayor de San Marcos, Lima, Peru; 4 Department of Veterinary Public Health and Food Safety, Ghent University, Ghent, Belgium; 5 Nicholas School of Environment and Duke Global Health Institute, Duke University, Durham, USA; 6 School of Veterinary Medicine, Universidad Nacional Mayor de San Marcos, Lima, Peru; 7 Center for Global Health, Universidad Peruana Cayetano Heredia, Lima, Peru; 8 Cysticercosis Unit, National Institute of Neurological Sciences, Lima, Peru; 9 School of Public Health, Oregon Health & Science University and Portland State University, Portland, USA

**Keywords:** *Taenia solium*, Agent-based modeling, Model calibration, Model transferability, Human taeniasis, Pig cysticercosis, Control intervention simulations, Infectious diseases modeling

## Abstract

**Background:**

Models can be used to study and predict the impact of interventions aimed at controlling the spread of infectious agents, such as *Taenia solium*, a zoonotic parasite whose larval stage causes epilepsy and economic loss in many rural areas of the developing nations. To enhance the credibility of model estimates, calibration against observed data is necessary. However, this process may lead to a paradoxical dependence of model parameters on location-specific data, thus limiting the model’s geographic transferability.

**Methods:**

In this study, we adopted a non-local model calibration approach to assess whether it can improve the spatial transferability of CystiAgent, our agent-based model of local-scale *T. solium* transmission. The calibration dataset for CystiAgent consisted of cross-sectional data on human taeniasis, pig cysticercosis and pig serology collected in eight villages in Northwest Peru. After calibration, the model was transferred to a second group of 21 destination villages in the same area without recalibrating its parameters. Model outputs were compared to pig serology data collected over a period of 2 years in the destination villages during a trial of *T. solium* control interventions, based on mass and spatially targeted human and pig treatments.

**Results:**

Considering the uncertainties associated with empirical data, the model produced simulated pre-intervention pig seroprevalences that were successfully validated against data collected in 81% of destination villages. Furthermore, the model outputs were able to reproduce validated pig seroincidence values in 76% of destination villages when compared to the data obtained after the interventions. The results demonstrate that the CystiAgent model, when calibrated using a non-local approach, can be successfully transferred without requiring additional calibration.

**Conclusions:**

This feature allows the model to simulate both baseline pre-intervention transmission conditions and the outcomes of control interventions across villages that form geographically homogeneous regions, providing a basis for developing large-scale models representing *T. solium* transmission at a regional level.

**Graphical Abstract:**

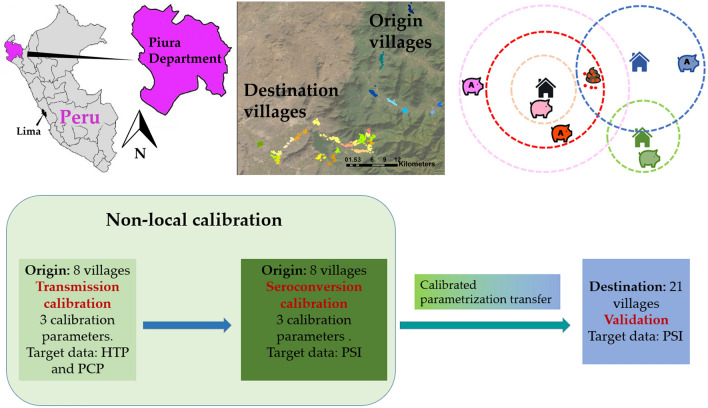

**Supplementary Information:**

The online version contains supplementary material available at 10.1186/s13071-023-06003-9.

## Background

*Taenia solium* is a zoonotic cestode parasite that infects human and pig hosts. In humans, most of the morbidity occurs when, after the ingestion of *T. solium* eggs, the resulting metacestode stage of this parasite infects the central nervous system with cysts (neurocysticercosis, NCC). NCC can lead to headaches, seizures, intracranial hypertension and sometimes death [1, 2]. Humans can also host the adult stage *T. solium* tapeworm in the intestines (human taeniasis, HT), acquired by ingesting cysts harbored in pig tissues. Pigs become infected with *T. solium* cysts (pig cysticercosis, PC) by ingesting human feces containing *T. solium* eggs produced by intestinal tapeworms in HT. This transmission cycle typically occurs in rural areas where pigs roam freely and have access to human feces due to a lack of sanitation infrastructure. Poor hygiene practices contribute to human NCC mainly through the fecal-oral route.

Three decades ago, *T. solium* was identified as a potentially eradicable parasite [3]. Although many control interventions since have been tested [4–9], most have achieved only modest reduction in transmission in the short term, with long-term effects remaining unknown because the interventions and/or monitoring typically have not continued beyond the end of the study. Short, resource-intensive interventions targeting elimination in relatively small geographic areas have achieved profound, short-term reduction in transmission [10, 11], but long-term evidence of sustained elimination effect is similarly absent. These limitations are primarily the result of resource limitations for addressing neglected tropical diseases, as the development and testing of interventions to increase both short- and long-term effectiveness and impact require significant investments of time and resources. Models of *T. solium* transmission and, more broadly, of helminth diseases [12], can aid in designing and testing new elimination or control intervention strategies in vitro, reducing the costs and time required for the transition from design to field implementation [13, 14].

To simulate the entire range of processes involved in the transmission of *T. solium*, models should encompass a broad range of temporal and spatial scales [15]. Multiple temporal scales are needed to simulate both short-term impacts and possible long-term resurgence, while multiple spatial scales are necessary to capture the different drivers of transmission. Small-scale spatial processes influence the formation of transient transmission hotspots around households with HT [16]. The distribution and density of sanitation facilities, free roaming pig-raising practices, land cover types and socioeconomic factors contribute to village-scale processes. Large-scale processes such as human movements and pig and pork trade between villages and urban centers also play a role in determining both village- and regional-scale transmission [17]. However, the design and use of multilevel *T. solium* models integrating both local- and large-scale dynamics are challenging because of the lack of similarly scaled real-world outcome data. Large-scale and longitudinal data on HT and PC are often not collected because of cost, complexity and the confounding effects that measurement may have on the system under study (HT must be treated when diagnosed).

To address these issues associated with large-scale models, we developed a non-local approach to the Bayesian calibration of CystiAgent, our agent-based model (ABM) of *T. solium* transmission [18]. This calibration methodology exclusively relies on non-local parameters that represent biological processes that should be invariant across villages with similar populations. An example of such parameters is the probability of acquiring HT infection upon ingestion of a single *T. solium* cyst. In contrast, village-specific or local parameters are incorporated in the model but are not subjected to calibration. For every new simulated village, the specific values of these local parameters, which are deliberately chosen to be easily and inexpensively collected, must be known.

Simulated and observed outcome data from a group of calibration villages are represented through vectors, and calibration performance is evaluated by comparing the distance between the vectors of observed and simulated data. Therefore, the process of non-local calibration looks for a model parametrization that is optimal not only for a single village but also for all the calibration villages together. This characteristic, coupled with the fact that the calibration parameters are all non-local and thus exportable from village to village, makes the resulting calibrated parametrization potentially spatially transferable to other villages outside the calibration group without any additional calibration.

In our first attempt to apply this non-local approach, we calibrated the model using HT and PC prevalences as target observables in three villages and then successfully transferred the calibrated model to five additional nearby villages [18]. This was an important initial step in evaluating this approach, but it was limited to a small number of villages located in a very small geographical area, and the validation of geographic transferability was based on a qualitative comparison between observed and simulated data collected during one single field trial. While replication using additional datasets and a more robust quantitative assessment are necessary to gain confidence with this calibration approach, appropriate datasets containing the outcomes of HT and PC prevalence from a large number of villages are not available. This barrier could potentially be addressed if our model could simulate pig exposure to the pathogen (in addition to PC infection) as a model outcome, as the prevalence of antibodies against *T. solium* in pigs has been measured in many cross-sectional and interventional studies [8, 19–21].

In this article, we extend our previously published ABM of local-scale *T. solium* transmission, CystiAgent [18, 22], by incorporating a new module to simulate the development of serum antibodies against *T. solium* in individual pigs. This model update allowed us to complete a more robust assessment of the non-local calibration method by validating CystiAgent against longitudinal datasets gathered during previously published community intervention trials that used pig seroprevalence as an outcome measure. We anticipate that this revised CystiAgent model, updated with the capacity to simulate pig exposure and with demonstrated geographical transferability among villages within a region, can serve as a foundational component in the development of multilevel models encompassing multiple villages and urban areas.

## Methods

### Overview of approach

As a first step we develop and introduce a new module into the ABM capable of representing the process of pig development of antibodies upon exposure to *T. solium* eggs or through maternal transmission. We then used existing datasets from two distinct previously published community intervention trials to calibrate the model and then to assess its transferability. The revised model was calibrated, following the non-local approach described above, using observed data of human taeniasis prevalence (HTP), pig cysticercosis prevalence (PCP) and pig seroincidence (PSI) obtained from eight rural villages located in the Piura region in northern Peru. The resulting calibrated parameters was then transferred to a second group of 21 geographically separate destination villages located in the same department. Baseline values and intervention impacts were simulated, and the pig seroprevalence data collected during an intervention trial in the destination villages were used to quantitatively validate both the spatial transferability of the model and the effect of control interventions simulated by the model.

### Model description

#### ABM short description

CystiAgent is an ABM that simulates *T. solium* transmission in a rural village. The model will be briefly described below while the details of the core structure are described elsewhere [18]. The model incorporates humans, pigs and households as agents, capturing the essential factors for *T. solium* transmission. It considers the geographical distribution of households, human and pig populations, and their behaviors. Human agents are associated with outdoor defecation sites around their households, which become contaminated if they carry a tapeworm. The degree of contamination depends on the availability and use of latrines. Pigs can become infected by coming into contact with contaminated sites within their roaming areas. The model calculates the number of *T. solium* cysts in infected pigs based on the level of contamination they are exposed to.

Both the human and pig populations are dynamic, reflecting natural rates of births, deaths, emigration and immigration. New human agents are periodically introduced to simulate migration or visits. The pig population aligns with observed herd size, slaughter age and import/export patterns in the region. Each household manages its herd size through export, sale or slaughter. When a pig is slaughtered at home, the resulting pork portions are distributed among the members of the household owning the pig as well as neighboring households, and subsequently consumed. Ingesting a *T. solium* cyst is associated with a probability of developing a tapeworm, and multiple tapeworm infections are not allowed. The model does not consider human cysticercosis or related seizure disorders. Minor modifications to the CystiAgent core are described in Additional file 1.

#### Pig seroconversion module

**Fig. 1:**
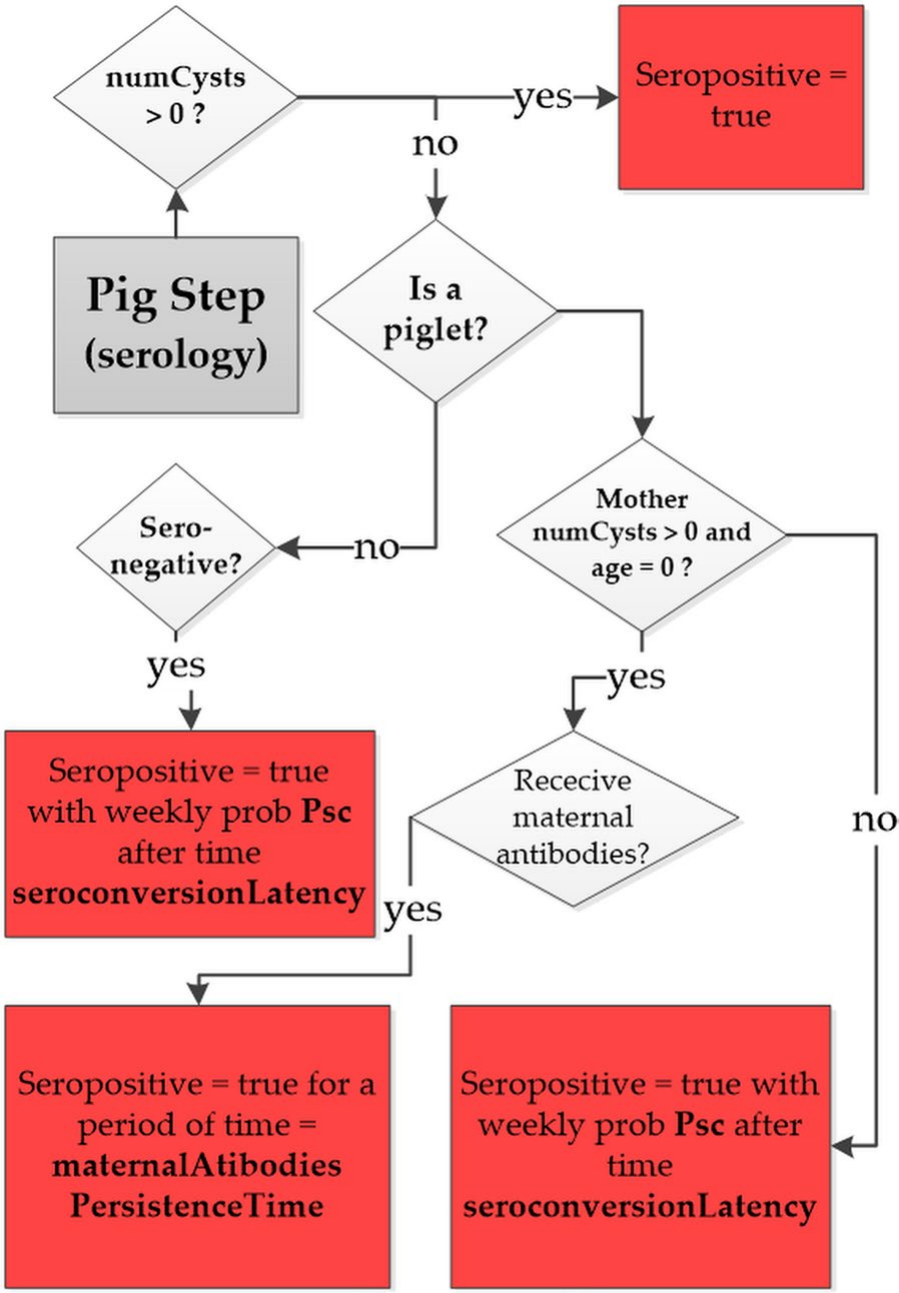
Flow diagram representing the process of development of antibodies against *T. solium* in pig. In the diagram numCysts is the number of cysts infecting the pig and Psc is the probability of seroconversion

To compare the simulated and observed values of PSI, a new module was added to the previous version of CystiAgent [18] to simulate the pig seroconversions process. In the model, a pig is considered seropositive if it has developed a level of antibodies that would result in a positive EITB assay according to the experimental setup described above. As depicted in the flow diagram in Fig. 1, the model accounts for four potential causes of serological state change in pigs: infection, exposure to *T. solium* proglottids or eggs and transfer of maternal antibodies.

The seroconversion is reversible only in the latter case, and the piglet returns to a seronegative state following weaning unless it has become positive through infection or exposure. The process of seroconversion through exposure is based on the calculation of *T. solium* contamination levels to which the pig is exposed during each model time-step (1 week). As described in [18], the levels of contamination from proglottids CP and eggs CE are assumed to be proportional to the number of defecation sites contaminated with proglottids and eggs within the pig roaming area, respectively. The factor *seroConvertPtoEFact* accounts for the lower density of *T. solium* eggs in a site contaminated with eggs compared to a site contaminated with proglottids. For each simulation time step and for each pig agent, the model counts the number of defecation sites contaminated with proglottids, eggs or both to which the pig is exposed. The resulting contamination levels are then inputted into an exponential dose-response model to calculate the weekly probability of seroconversion (Psc(*t*)) for each pig during the timestep *t*:

1 $$\mathrm{Psc}\left(t\right)= 1- {e}^{-\mathrm{seroConvert} \bullet \mathrm{CT}\left(t\right)},$$

where $\mathrm{CT}\left(t\right)=\mathrm{ CP}\left(t\right)+\mathrm{seroConvertPtoEFact }\bullet \mathrm{ CE}(t)$ represents the total contamination to which the pig is exposed during the time step t and *seroConvert* is the exponential dose–response model factor. If exposure results in seroconversion, the pig’s serological state changes to seropositive after a latency period of 2 week (as specified by the model parameter: “*seroconversionLatency*”). The process of seroconversion through maternal antibody transfer is represented as follows: when piglets are born to a PC-infected sow, they acquire maternal antibodies and become immediately seropositive with a probability specified by the model parameter “*propPigletsMaternalProtection*,” regardless of the mother’s cysts burden. The piglet’s seropositive status is maintained for a period of 14 weeks [23], after which the piglet seroconverts to seronegative. During this period, the piglet is exposed to *T. solium* environmental contamination and, like any other pig agent, can seroconvert through exposure with a weekly probability $\mathrm{Psc}(t)$. The parameters introduced in the seroconversion module of CystiAgent are presented in Table 1. Three of these parameters were selected for the purpose of calibrating the model.

**Table 1:** Seroconversion module model parameters

Parameter name	Value	Notes	References
seroconvert	(^a^)		Calibration parameter
seroConvertPtoEFact	(^a^)		Calibration parameter
seroconversionLatency	2 weeks	Latency of seroconversion after exposure	[24]
propPigletsMaternalProtection	(^a^)		Calibration parameter
maternalAntibodies-PersistenceTime	14 weeks	Persistence of maternal antibodies in piglets after birth	[23]

^a^Value obtained through calibration

### Origin and destination field trial datasets

**Fig. 2:**
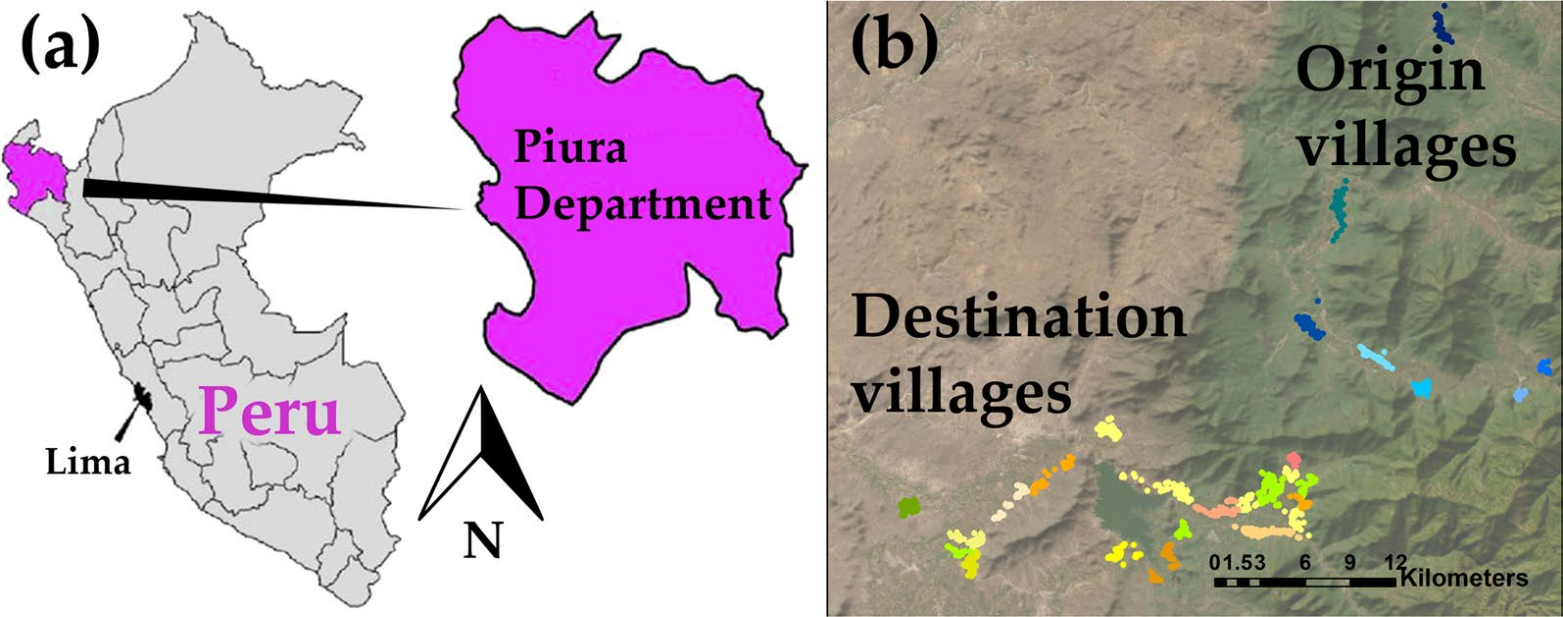
Study area maps; **a** the Piura region in north Peru; **b** the relative location of the 8 origin villages and the 21 destination villages. Each dot on the map represents a different household. Households from the same village are in the same color. Households from origin villages are showed in shades of blue while the households from destination villages are represented in shades of green, orange and yellow

In this study, two separate datasets are used. The first dataset (referred to as the “origin dataset”) serves as the calibration set, while the second dataset (the “destination dataset”) is used to evaluate the spatial transferability and the simulated intervention effects. These datasets stem from two distinct community cluster randomized trials conducted in rural villages situated in two separate areas of the region of Piura in the northwest region of Peru (Fig. 2). In these rural communities, pigs are allowed to roam freely, access to proper sewage and latrine systems is limited, and open defecation practices are prevalent, leading to exposure of pigs to human feces and hence to the creation of an optimal environment for the transmission of *T. solium* [11].

The origin trial [4, 5], conducted between 2014 and 2015, aimed at evaluating the impact of education and increased community surveillance on *T. solium* transmission in eight villages. The trial resulted in a comprehensive household census and in the collection of blood samples from all pigs older than 6 weeks over four rounds. At the end of the trial, the HTP and PCP were determined through mass human stool screening and pig necroscopy, respectively. No significant effects of increased community surveillance were observed at the end of the trial period, so all the data collected can be considered representative of the baseline endemic level of all enrolled villages.

The destination dataset is derived from a field control intervention trial conducted from 2015 to 2017 [8]. Out of the 23 villages included in the trial, data from 21 villages were used to create the destination dataset, while the remaining two villages were excluded because of the small pig population (7 pigs) or the absence of significant seroprevalence at the baseline pre-intervention stage (no transmission ongoing) [25]. Analogously to the origin trial, a baseline household census was conducted to gather information relevant to *T. solium* transmission. The villages were randomly assigned to six different intervention arms each with a different *T. solium* control strategy. Three of these arms focused solely on human intervention: Mass Treatment (Mass Trt), Ring Treatment (Ring Trt) and Ring Screening (Ring Scr). The remaining three arms targeted both humans and pigs: Mass Trt (P), Ring Trt (P) and Ring Scr (P). In the Mass Trt strategy, all village residents ≥ 2 years old received a single dose of niclosamide every 6 months for a total of five treatment rounds. The Ring Trt involved conducting an active surveillance based on pig tongue inspection [26, 27], every 4 months for seven rounds. If cysticercosis was found in the tongue of a pig, a 100-m treatment ring was established around the household owning the pig. All members ≥ 2 years old from households within the ring were treated with two oral doses of niclosamide separated by 15 days. All the tongue-positive pigs were either purchased or treated with a single dose of oxfendazole. In the Ring Scr approach, the same active surveillance based on pig tongue inspection was conducted. Upon identifying a ring, stool samples were requested from villagers ≥ 2 years old residing within the ring and tested for *Taenia* spp. eggs or antigens. Individuals diagnosed with HT were offered a single dose of niclosamide treatment. The Mass Trt (P) strategy added pig treatment to human treatment with seven rounds of mass pig treatment occurring every 4 months. In Ring Trt (P) and Ring Scr (P) strategies, only pigs aged > 5 weeks within the identified rings were treated. The primary outcome of the trial was the determination of the PSI in all pigs aged > 5 weeks and born during the two year study through 7 pig serosurveys conducted every 4 months [8]. The secondary outcome of the study was HTP, which was determined offering presumptive niclosamide treatment at the study end to all residents ≥ 2 years old and collecting and testing stool samples from all treated individuals.

Pig serological outcome data were based on the enzyme-linked immunoelectrotransfer blot (EITB), which detect antibodies against *T. solium* cysticercosis in pigs [28]. Reaction to any of six glycoproteins, GP39/42, GP24, GP21, GP18, GP14 or GP13, was considered a positive result. The GP50 band was not considered because of the known cross-reaction with *Taenia hydatigena*, a co-endemic and highly prevalent infection of pigs [29].

### Model calibration

**Fig. 3:**
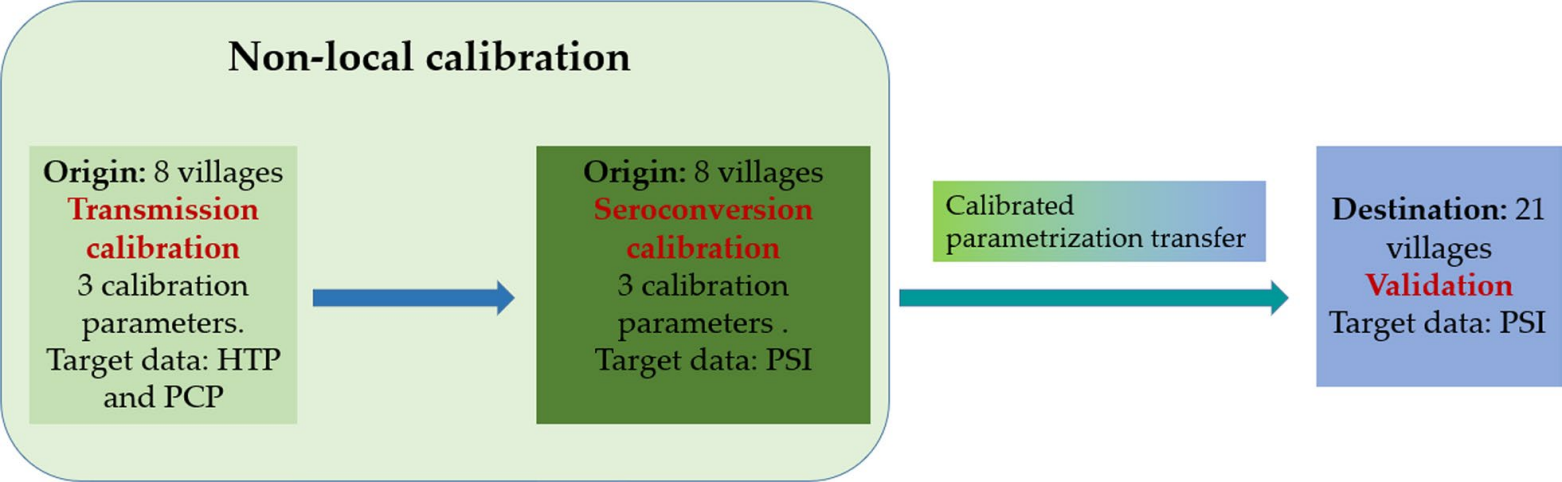
Model calibration and validation scheme

The CystiAgent model was calibrated and validated based on empirical observations of HTP, PCP and PSI as target data in this study. In the model there is not an explicit representation of immunity in pigs. As result, pig seroconversion has no impact on the transmission process. Hence, as illustrated in Fig. 3, the calibration process was divided into two distinct stages: transmission calibration followed by seroconversion calibration. Model parameters were separated in two categories: local and non-local. As shown in Table 2, six non-local parameters were selected as calibration parameters. These parameters are connected with the processes of human and pig infection and pig seroconversion. Table 2 also shows the local parameters that vary from village to village in this study. The rest of parameters were kept constant across different villages.

**Table 2:** Local parameters that vary from village to village and non-local parameters used to calibrate the model

Parameter name	Description
Non-local	
pHumanCyst	Probability of human HT upon ingestion of a single *Taenia solium* cyst. Transmission calibration parameter
pigProglotInf	Average number of cysts infecting a pig through a weekly exposure to one defecation site contaminated with *T. solium* proglottid. Transmission calibration parameter
pigEggsInf	Average number of cysts infecting a pig through a weekly exposure to one defecation site contaminated with *T. solium* eggs. Transmission calibration parameter
seroconvert	Parameter of the dose–response exponential model representing the process of pig seroconversion. Seroconversion calibration parameter
seroConvertPtoEFact	Parameter that accounts for the lower density of *T. solium* eggs in a site contaminated with egg compared to a site contaminated with proglottids. Seroconversion calibration parameter
propPigletsMaternalProtection	Proportion of infected sows that pass *T. solium* antibodies to their offspring. Seroconversion calibration parameter
Local	
Household distribution	Geographical location of each village household
Household	Number of people in each village household
Household pig composition	Households owning pigs in the village and number of owned villages
Latrine	Distribution of latrine in the village among the village households
Corral	Distribution of corrals in the village households
corralUse	Use of corral among pig-owner households (always, sometimes, never)

We applied the sequential Monte Carlo (SMC) approach to the likelihood-free method of approximate Bayesian computation (ABC), as described in [18, 30] for model calibration. We used a non-local approach to calibration [18]. The SMC method involves generating a sequence {εi} of decreasing ABC tolerances. For each tolerance εi, a straightforward ABC rejection sampler is executed, followed by exploration of the parameter space using importance sampling guided by the posterior distribution obtained in the previous stage. The initial rejection sampler begins with a uniform prior distribution of calibration parameters, spanning a wide and reasonable range of parameter values (see Additional file 1). The ABC distance function was defined as a normalized Euclidean distance between the observed and simulated vectors of target values for the 8 calibration villages [18]. During the calibration process, the first ABC-SMC stage involved 120,000 sampling points each corresponding to a different combination of calibration parameters. The second stage used 60,000 sampling points, the third stage used 40,000 sampling points, and the fourth stage used 30,000 sampling points. From the first to the fourth stage, only one simulation per sampling point was performed. The tolerance of each stage was set to accept the 20 sampling points that produced the lower values of the distance between observed and simulated vectors of target data. This tolerance was chosen as a trade-off between the convergence speed of ABC-SMC and the accuracy of sampling in the calibration vector space. In the fourth stage, both for transmission and seroconversion calibrations, the process converged as the distance between observed and simulated data for the best performing sampling point did not decrease compared to the third stage. To mitigate incorrect acceptance of sampling points due to the fluctuations of model output, after the first four stages we conducted six additional ABC-SMC rounds of 5000 points each. In these rounds, for each sampling point the simulations were repeated eight times and the average of the results from these eight repetitions was used to calculate simulated target data.

### Estimation of empirical errors in the destination dataset and assessment of model transferability

The model was considered to have been successfully transferred to a destination village if the simulated value of PSI for that village fell within the error interval defined by twice the standard deviation (SD) of the time series of observed PSI values in that village. A direct calculation of PSI variance for the destination dataset is not feasible because of the absence of longitudinal observations in this trial. However, it is possible to hypothesize that intra-village variances are homogeneous across both the origin and destination villages, provided that this homogeneity holds true for the origin villages. This hypothesis of intra-village variance homogeneity across the origin villages can be tested using a Fligner-Killen test [31]. The resulting *p*-value of 0.722 indicates that intra-village variances are indeed homogeneous across the origin villages. To estimate the intra-village variance from the entire origin dataset, we considered the following random effect model:

2 $${\mathrm{PSI}}_{\mathrm{Vp}}= \mu +{U}_{\mathrm{V}}+{W}_{\mathrm{Vp}}.$$

The observed ${\mathrm{PSI}}_{\mathrm{Vp}}$ of village *V* is expressed as the sum of µ that is the average PSI value across all villages, *U*_V_ a random effect to account for inter-village variability and *W*_Vp_ a random term accounting for intra-village variability due to individual pigs. The corresponding total variance σ_VW_ will be expressed as:

3 $${\sigma }_{\mathrm{VW}}= {\sigma }_{\mathrm{V}}+{\sigma }_{\mathrm{W}},$$

The variances σ_V_ of the *U*_V_ term and *σ*_W_ of the *W*_Vp_ term represent the inter- and the intra-village variances associated to empirical PSI, respectively. This formulation assumes that the measurements of porcine cysticercosis at successive time points are uncorrelated, which may not be the case if the time interval between measurements is too short. If the time points are correlated, then we are underestimating the standard deviation. The random model was fit using the lmer module of the R package lme4 [32].

### Simulation of spatially targeted interventions

As noted, CystiAgent explicitly represents the geographic space of simulated communities. As a result, not only can the observed spatial clustering of the parasite [16] be replicated by the simulations, but the model also allows for the simulation of spatially targeted interventions aimed at reducing the parasite burden in selected transmission hotspots. This is essential for simulating the effect of interventions based on the ring strategy adopted in the destination dataset. The ring interventions are simulated by the model exactly replicating the experimental protocol described in [8]. For Mass Trt and Mass Trt (P) interventions, humans and pig agents in the appropriate age segments were selected at random in the village and then treated. For Ring Trt, Ring Trt (P), Ring Scr and Ring Scr (P) intervention strategies, pigs were selected at random for tongue inspection (see the Table 3 for tongue inspection sensitivity and specificity used in the model). For each tongue-positive pig, a 100-m ring was opened, and humans were screened and/or treated accordingly. The efficacies of human niclosamide and pig oxfendazole treatments used in the model are shown in Table 3. The participation rates for pig tongue inspection, human stool screening and human treatment were set to be equal to the corresponding participation rates in the field trials for each round of each village.

**Table 3:** Model parameters used to simulate the destination dataset interventions. nC is the number of *Taenia solium* cysts infecting a pig

Parameter name	value	Notes	References
TongueSensi1000	1.0	Tongue inspection sensitivity for nC ≥ 1000	[26]
TongueSensi100	0.91	Tongue inspection sensitivity for 100 ≤ nC < 1000	[26]
TongueSensi10	0.55	Tongue inspection sensitivity for 10 ≤ nC < 100	[26]
TongueSensi1	0.23	Tongue inspection sensitivity for 0 < nC < 10	[26]
TongueSpeci	0.025	Tongue inspection specificity	[26]
elisaSens	0.97	Sensibility of coproantigen ELISA	[33]
NiclosamideTreatEff	0.86	Efficacy of niclosamide treatment	[34]
oxfTreatEff	1.0	Efficacy of oxfendazole treatment	[35]

### Model software and simulations setup

The model was implemented using the MASON platform [36], a free, Java-based, discrete-event multi-agent toolkit, together with our in-house Java code for ABC calibration. Each village simulation started with 3500 burn-in time steps. The results reported in this study were obtained from 128 repeated simulations for each parametrization of the model. Within each simulation, the observables of interest were sampled 100 times at discrete points in time with a separation of 100 weekly time step. The simulations were run on the Exacloud Cluster at the Oregon Health and Science University Advance Computing Center, USA.

## Results

### Estimation of empirical data error intervals

We fitted the random effect model to empirical PSI data and obtained the inter- and intra-village variances for the origin dataset, which were 0.018 and 0.01, respectively. Using the intra-village variance, we estimated the standard deviation of PSI to be 0.1 with a 95% CI of (0.07, 0.15). The inter-village standard deviation was estimated to be 0.13 with a 95% CI of (0.07, 0.24). We calculated the empirical error associated with PSI by considering a 2 SD interval around the observed value, which was therefore ± 0.2. Based on the assumption of intra-village variance homogeneity, we applied the same empirical error to all destination villages for both baseline pre-intervention and post-intervention data.

### Model calibration using the origin dataset

**Fig. 4:**
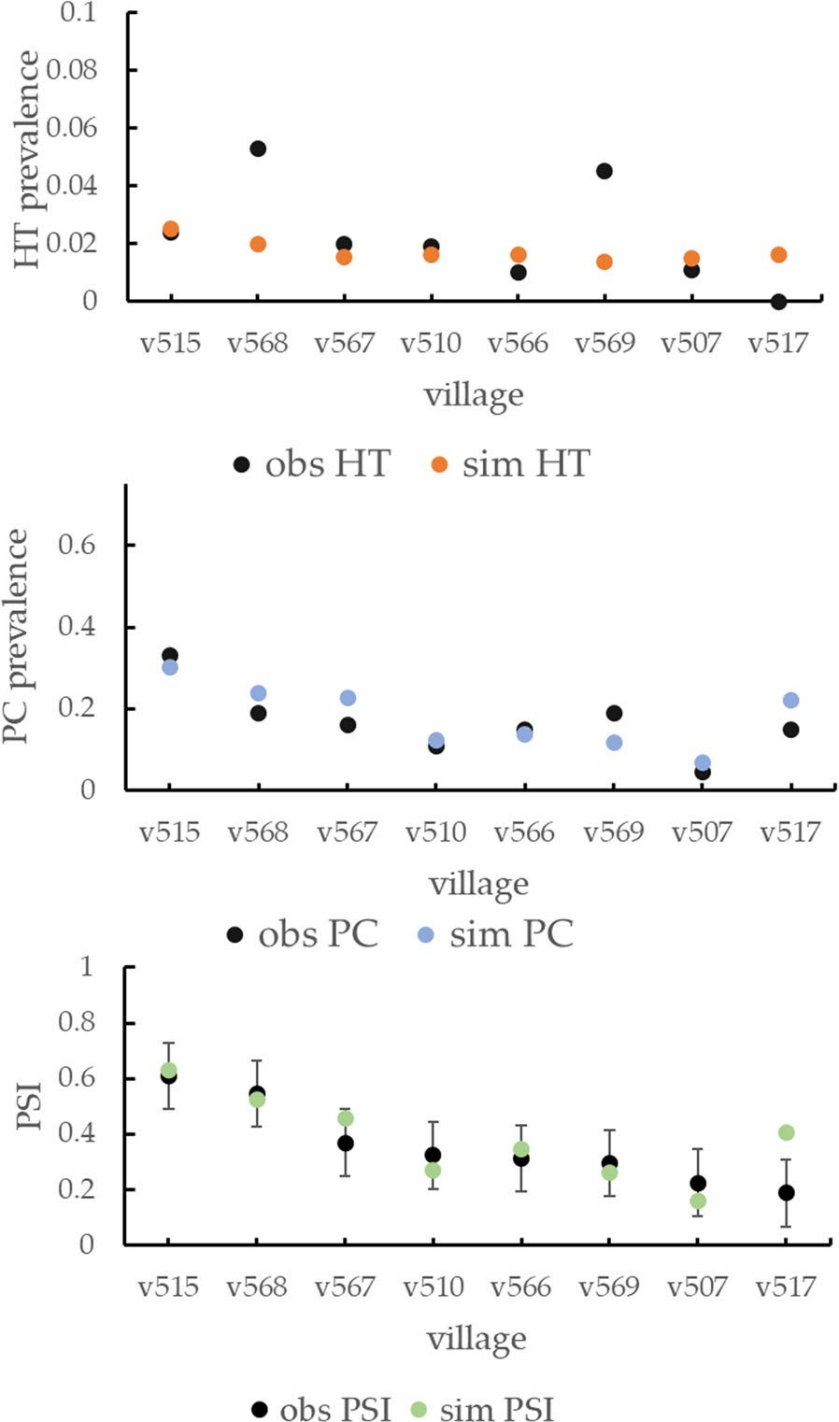
HTP, PCP and PSI, observed (obs) and resulting from simulations (sim), using the calibrated model parametrization for the 8 villages composing the origin dataset, and confidence intervals around PSI values

Table 4 shows the transmission parameter values resulting from calibration, which were obtained from the tenth sequential Monte Carlo sampling round [18] selecting the sampling point corresponding to the minimum distance between observed and empirical HTP and PCP data of origin villages [18]. The simulated HTP and PCP corresponding to the calibrated parametrization are shown in Fig. 4 along with the corresponding observed data. The average value over the eight origin villages of simulated HTP was found to be similar to the average empirical observation (average values: 0.017 simulated, 0.023 observed), with a much lower standard deviation for inter-village variations (SD: 0.0034 simulated, 0.017 observed). The values of PCP showed that the averages were more similar (average values: 0.18 simulated, 0.17 observed), as well as the standard deviations (SD: 0.074 simulated, 0.076 observed).

**Table 4:** Tuned values of calibration parameters for transmission and seroconversion module

Parameter	Calibrated value	Posterior distribution range
Transmission		
pHumanCyst	2.41 10^–4^	[2.3 10^–4^, 2.5 10^–4^]
pigProglottidInf	8.05	[7.38, 8.54]
pigEggsInf	0.84	[0.82, 1.05]
Seroconversion		
seroconvert	2.85	[1.11, 3.16]
seroConvertPtoEFact	0.023	[0.014, 0.78]
propPigletsMaternalProtection	0.86	[0.45, 0.98]

The second step of the calibration process involved tuning the parameters related to pig seroconversion. Table 4 presents the calibrated parameter values, which were obtained after 10 rounds of ABC-SMC. Figure 4 depicts the simulated PSI along with the corresponding observed data and associated error bars. As the observed PSI values correspond to the average of three separate measurements at three different time points, the standard errors were obtained by dividing the standard deviation from the random effect model fit by the square root of 3. When comparing the simulated and observed PSI using these errors, we find that the simulated PSI are consistent with the observed values for seven out of eight villages (88%). The average simulated PSI value across all origin villages was slightly lower than the average empirical observation (average values: 0.38 simulated, 0.36 observed) but with the same standard deviation (SD: 0.14 for both simulated and observed data).

### Transfer of the model to the destination dataset

Data from all 21 villages in the destination dataset were used to validate the transfer of the calibrated parameterization from the origin villages. As previously described, the destination dataset contains both pre-intervention and intervention data. Since the origin field trial did not involve any interventions, none of the intervention parameters presented in Table 3 were adjusted during the calibration process. Therefore, the validation process for the destination simulations can be conceptually separated into two distinct parts: pre-intervention validation and intervention validation. The former entails comparing empirical pre-intervention destination data with the simulation outputs generated by the model parameterization obtained from the origin calibration process. Thus, pre-intervention validation can be considered as the validation of the spatial transfer of the model from the origin to the destination dataset. The latter part adds an additional component to the validation of the spatial transfer: the validation of simulated effects of all intervention strategies in the destination trial.

### Pre-intervention validation

**Fig. 5:**
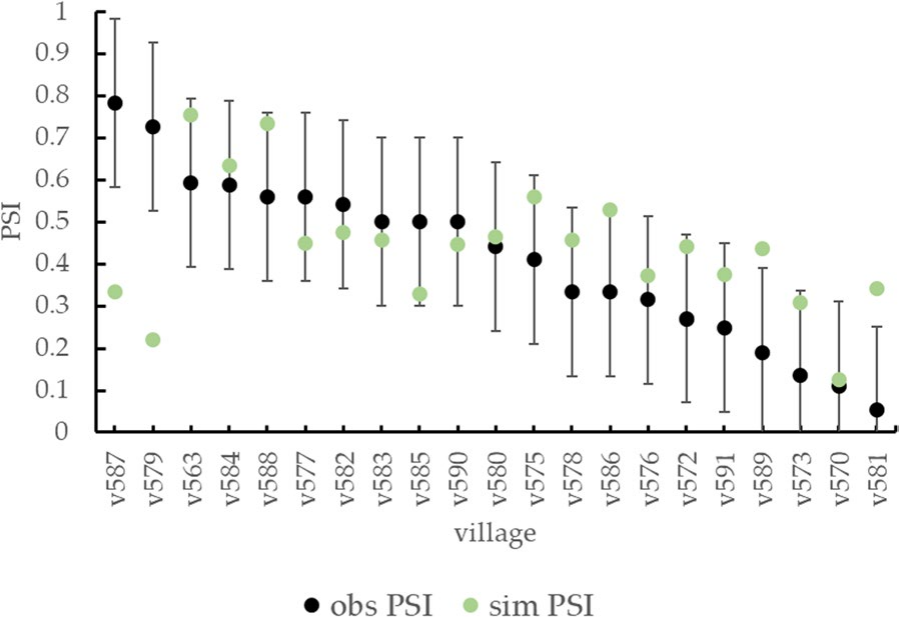
Pre-intervention PSI for the 21 destination villages observed (obs) and resulting from simulations (sim). The observed data are represented together with their empirical error calculated as described in the methods section

The pre-intervention PSI observed in the 21 destination villages (Fig. 5) had an average value of 0.41, indicating moderate endemism for the selected region of the destination trial. However, the PSI values observed in each destination village indicated a wide range of values (SD: 0.19), ranging from 0.05 in village v581, which is typical of almost zero transmission [25], to 0.78 in village v587, reflecting much more intense infection and exposure of pigs. The simulated PSI values were compared to the corresponding destination trial data (Fig. 5) to validate them and assess the model's spatial transferability. This comparison revealed that the average seroprevalence across the 21 villages was very similar (0.44) in the simulation compared to observed values (0.41). Furthermore, for 17 of the 21 (81%) destination villages, the simulations produced a PSI value that was within the accepted error interval (computed using 2 SDs without dividing by the square root of 3 given that we have only one observation in destination villages) of the corresponding empirical data. Among the villages with PSI value outside the accepted error interval, two, at the higher end for observed seroprevalence values, produced simulated values that overestimated the empirical data (villages v581, v589), and two villages (villages v579, v587), among the lowest observed seroprevalence values, showed simulated values that underestimated the observed data. The variability of simulated PSI among destination villages was 23% lower than the observed variability (simulated SD: 0.15), with maximum and minimum simulated PSI values of 0.75 (village v563) and 0.13 (village v570), respectively.

### Intervention validation

**Fig. 6:**
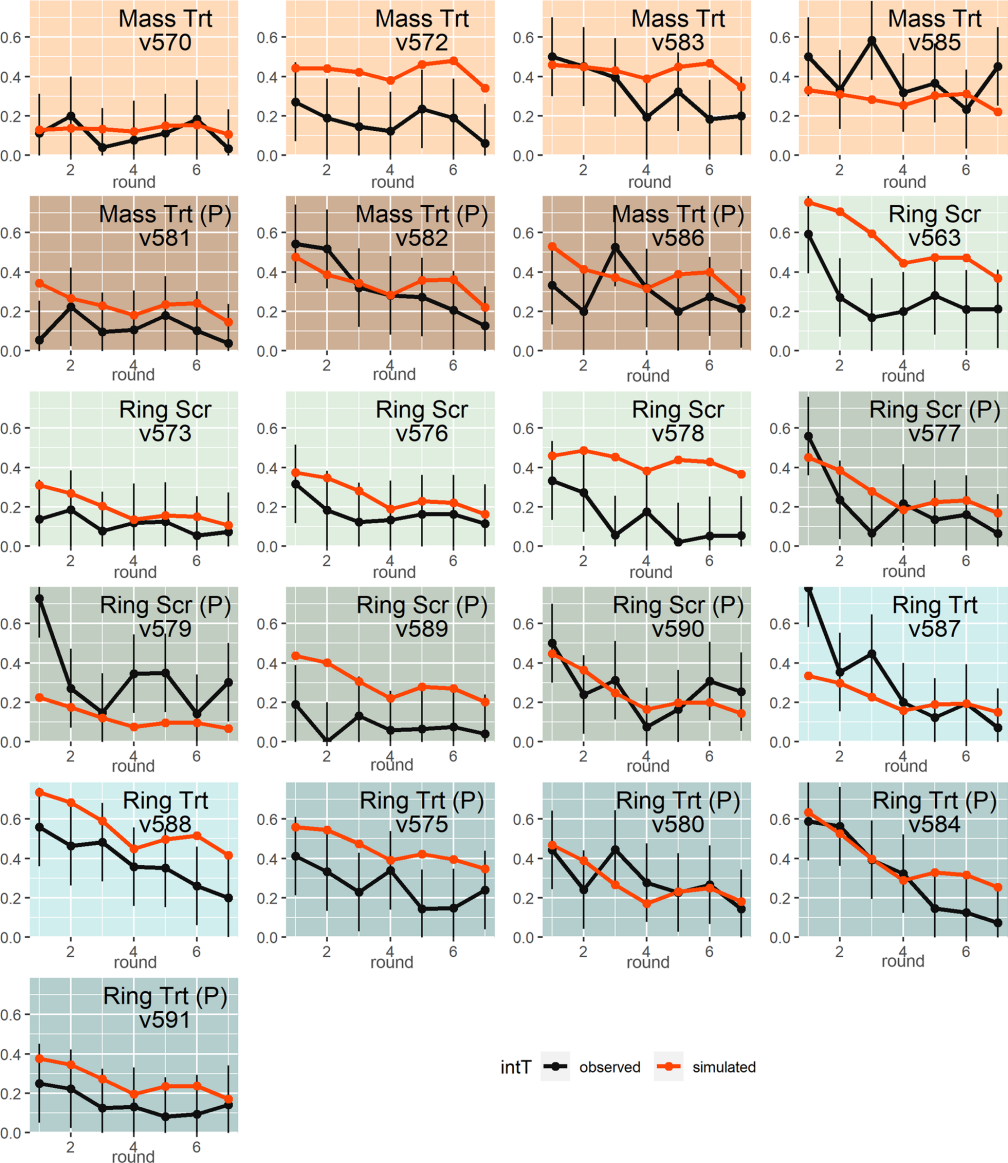
Plots of observed (black curves) and simulated (red curves) PSI for each of the 21 villages composing the destination trial. The data for each village are presented in a separated plot using a color identification to distinguish the different intervention strategies applied to the village: salmon for Mass Trt, brown for Mass Trt (P), light green for Ring Scr, green for Ring Scr (P), light blue for Ring Trt, dark blue for Ring Trt (P)

As shown in Fig. 6, although interventions produce clear effects on observed data, the curves resulting from the seven rounds of field PSI observations show a remarkable degree of noise due to fluctuations. For more than one village, high fluctuations bring the observed PSI back above the pre-intervention level. This occurred in village v570 round 2, village v585 round 3, village v581 round 2, village v586 round 3, village v573 round 2 and village v580 round 3. All these villages, with the exception of villages v573 and v580, were included in the Mass Trt and Mass Trt (P) intervention arms. As a general remark about empirical PSI curves, in the ring intervention arms, a pronounced decrease is observed during the first three or four rounds, especially for villages showing a high pre-intervention PSI, such as v563, v577, v579 and v587. The same pronounced decrease was not observed for mass treatment villages, for which the PSI seems to decrease more linearly.

Concerning simulations, in total, 92 out of 126 intervention measurements (73%) were validated, excluding pre-intervention values (Fig. 6). Concerning the last PSI measurement, the post-intervention PSI value, 16 out of 21 villages (76%) showed validated values. The destination villages with the lowest number of validated intervention points including pre-intervention were: v572 and v578 with 1 validated point and villages v563, v579 and v575 with 3 validated points. As a general remark, it can be observed that for eight villages, the model not only reproduces the pre-intervention PSI value but also the values of the following six intervention rounds. This is the case for villages v570, v582, v573, v576, v590, v580, v584 and v591. For other villages (villages: v581, v586), the pre-intervention or starting PSI levels are not reproduced, but the following values are. For no village did the simulations fail to produce a single validated point.

**Fig. 7:**
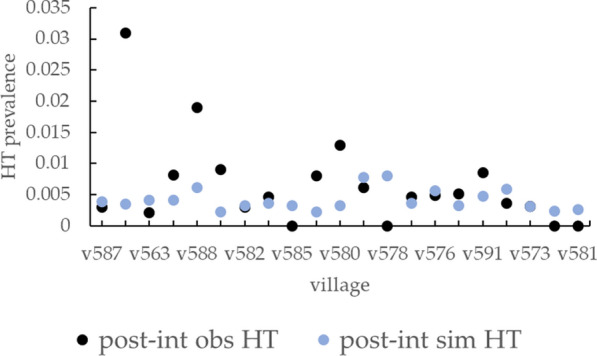
Post-intervention HTP observed (obs) and resulting from simulations (sim) calculated using the exported model parametrization for the 21 villages composing the origin dataset. The post-intervention HTP corresponds to the prevalence observed in the destination villages during the last round of interventions

Post-intervention HTPs, measured as a secondary outcome during the final round of the destination intervention trials, are compared with the simulated data in Fig. 7. The final round corresponds to the fifth and the seventh intervention rounds of Mass Trt villages and of Ring Scr or Ring Trt villages, respectively. For most villages, the simulated values appear to represent a good approximation of the empirical data, but uncertainties associated with observed HTP data are unknown, so quantitative comparisons are not possible. Villages v579 and v588 revealed very high post-intervention HTP, which is not replicated by simulated data. Several villages (v570, v578, v581, and v585) are associated with a zero post-intervention observed HTP, while for no village as for PSI values, simulations produced a zero HTP. The resulting average observed HTP across the destination villages is 6.5 × 10^–3^ compared with an average simulated HTP of 4.1 × 10^–3^. As for PSI data, the observed HTP prevalence showed a higher variability among destination villages, with a 7.1 × 10^–3^ standard deviation compared to the 1.6 × 10^–3^ of simulated PSI.

## Discussion

This article presents the first attempt to validate the regional spatial transferability of a fully non-local ABM of *T. solium* transmission used to simulate control intervention. In a first step, the model was calibrated against empirical data of HTP, PCP and PSI derived from a community trial that was conducted in eight villages in the Piura region of the northwestern Peru. In a second step, the calibrated model was spatially transferred to villages located in a second area of the same Piura region to be validated against pig seroincidence data collected during a cluster-randomized trial of *T. solium* control interventions that was conducted to study the effectiveness of the spatially targeted interventions. The model transfer proved successful, since the calibrated ABM model was able to generate validated pre-intervention outcomes for most of the destination villages. Intervention and post-intervention data were also validated for most villages, demonstrating that the model can correctly reproduce the effect of a wide range of *T. solium* control intervention types.

In this study, simulating pig seroincidence data was crucial to compare the observed and simulated data of the origin and destination datasets. We implemented a new module in CystiAgent that represents pig seroconversion based on a mathematical dose-response approach where the probability of pig seroconversion is expressed as a function of the doses of *T. solium* proglottids and eggs to which the pig is exposed. Dose-response modeling has been widely used to determine the probability of infection upon exposure to different levels of pathogens in human and animal diseases [37] and to study the onset of immunity upon exposure to different doses of vaccines [38]. Although more sophisticated dose-response models, such as the exact beta-Poisson, are available [39], we have chosen to use a simple stochastic exponential model to limit the number of calibration parameters, as all the parameters of any dose–response model are unknown. It is important to note that the ABM does not use a dose-response model for the pig infection process, using instead a representation based on the number of *T. solium* cysts that infect a pig following each exposure event. In addition to seroconversion for infection and exposure, the model includes a third component to seroconversion: the development of seropositivity for maternal antibodies transfer [40]. Although the process of maternal antibodies is parameterized based on limited knowledge and a scarce amount of data, we consider the current version to be a first step toward a more complete module that can be developed when new data on the piglet seroconversion process become available.

Regarding calibration results, the calibrated model parameters can effectively reproduce the observed PCP data from the origin trial for the majority of villages. The average values across the entire group of origin villages and the standard deviation of simulated PCP values are quite similar to empirical values. A qualitative comparison indicates that the model is able to reproduce the specific characteristics of each village, such as the particularly high PCP values in village v515 and the low value in village v507. The calibrated model does not appear to reproduce observed HTP values with the same precision as in the case of PCP. This outcome was somewhat expected because, in a low to moderate prevalence setting like the rural areas of Peru, the observed values of HTP vary considerably with changes in the number of tapeworm carriers in a village. This number can fluctuate significantly because of new infections and recoveries. Moreover, a substantial portion of the small group of village tapeworm carriers may not participate in the field trial screening activities producing a considerable alteration of the resulting observed HTP value. The effect of these fluctuations is particularly evident when comparing observed HTP and PCP values across origin villages. Figure 4 illustrates that for some villages, the observed HTP values do not seem coherent with the corresponding PCP values. For instance, village v517 shows an HTP of zero, which contrasts with the observed PCP value of 0.15 (average PCP across the origin group: 0.17). On the other hand, village v568 and v569 exhibit particularly high HTP values (0.053 and 0.045, respectively) despite observed PCP values that fall very close to the dataset average (v568: 0.19 and v569: 0.19). Villages with HTP values that do not align with the observed PCP values also exhibit a poor agreement between empirical and simulated HTP values (v517, v568, and v569). Compared to HTP, the observations of PCP are calculated over a higher proportion of pig populations, which gives the observation a lower uncertainty.

We note that the tuned values of transmission calibration parameters obtained by calibrating the model against the origin dataset HTP and PCP are not identical to those shown in a previous study [18], even though the field trial used to calibrate the model in both studies was the same. The reason for this change is related to several factors. First, the model was changed regarding the version presented in the previous study as described in Additional file 1. Second, in the previous study, only three villages of the trial were used to calibrate the model, while in this study, we used all eight villages, which should improve the quality of the calibration.

PSI measurements for the origin dataset were repeated three times, allowing for a more quantitative analysis using average observed PSI values to compare observed and simulated data. Using the standard deviation estimation divided by the square root of 3 as observed data standard error, we found that for seven of eight (88%) villages, the calibrated model parametrization produced a validated PSI value. The only non-validated village is, again, village v517, which showed the anomalous values of zero HTP. As a general remark, the simulated values of PSI for the origin villages seem to follow the features of observed data. The high values of villages v515 and v568 are well reproduced, as well as the low value of village 507.

Empirical PSI values of the destination villages are limited to one observation in time, which makes the effect of uncertainties particularly evident. Our findings revealed that the variability in observed pre-intervention data across villages was larger than that of simulated data. Given the high levels of fluctuations in observed values within villages, which we referred to as intra-village variance (*σ*_W_), one would expect that the total variance *σ*_VP_ of data observed at one point in time to be higher than the village-specific variance, which we referred to as inter-village variance (*σ*_V_), following equation ${\upsigma }$ VW $= {\sigma }_{\mathrm{V}}+{\sigma }_{\mathrm{W}}$ (3). In the case of destination dataset, the total standard deviation SD_VW_ for observations taken at one point in time is 0.19, and the intra-village SD_W_ for fluctuations over time is 0.1, we would expect then the inter-village SD_V_ to be: $\sqrt{{0.19}^{2}-{0.1}^{2}}=0.16$. Meanwhile, the standard deviation in the simulated values, which is equal to the inter-village variance because the simulated intra-village variance can be considered negligible since the simulated values averaged over many simulations, was 0.15, only slightly lower than what we found for the observed inter-village standard deviation. Further possible contributors to differences between the observed and simulated village seroprevalence variability include the impact of some of the specific characteristics of simulated villages, such as different rate of travels to and from external destinations or different attitudes toward *T. solium* in different villages, which are not represented in the model as we did not have village-specific measurements for these. Overall, there is good agreement not simply in average seroprevalence values but also in the expected inter-village variability of observed and simulated village averages.

As noted, several destination villages exhibit observed PSI value during interventions that are well above the pre-intervention level two or three rounds after the start of the intervention when a pure decreasing trend would be expected because of the nature of interventions applied. This behavior can be attributed to several causes. First, as previously mentioned, fluctuations in the number and location of tapeworm carriers in the village can lead to fluctuations in pig exposure to *T. solium* eggs and proglottids, resulting in changes in seroprevalence in the pig population. Similarly, fluctuations in pig populations due to pig turnover, such as during holidays when pig slaughtering rates are high, can produce sharp fluctuations in pig seroprevalence. Other factors that may have triggered variations are extreme events related to climate, human movements and pig or pork trades.

Since empirical PSI data are subject to high fluctuations over time in this study, the comparison between observed and simulated PSI data was made considering the empirical uncertainty given by such PSI fluctuations. The comparison of observed and simulated PSI resulted in a slightly lower proportion of validated results (73%) for intervention compared to pre-intervention observations (81%). The lower precision of the model in reproducing the intervention effects may be attributed to multiple causes. First, while we expected the SD in the destination villages, before intervention, to be similar to that in the origin villages, it may not be the case post-intervention. Indeed, fluctuations are expected to be somewhat higher at very low prevalence levels, hence using the same SD for pre- and post-intervention results should lead to a lower level of validation post-intervention. Additionally, the model module designed to simulate interventions was not calibrated at all as no intervention was included in the original dataset. As a result, some of the parameter values used to simulate interventions may be slightly inaccurate. However, overall, the decreasing trend along the screening rounds produced by the intervention appears to be reproduced by the model.

Despite the good coherence found in this study between simulated and observed values, the large fluctuations in PSI observations represent a significant limitation for the appropriate calibration of the model and the validation of its geographical transferability as confidence intervals around observed values remain wide, even though fluctuations in seroprevalence are expected to be lower than fluctuations in HTP. This is an intrinsic limitation which makes it difficult to separate the contribution from the background noise from the component associated to the true dynamics of *T. solium* transmission. Another limitation of the validation is that the groups of origin and destination villages, although further away from one another than in our prior work [18], were selected from a moderately restricted area which, for this reason, presents a certain degree of homogeneity. Adjustments may be necessary when transferring the model between two more dissimilar regions.

In the ABM used in this study, there are many local parameters that directly or indirectly contribute to determining the level of transmission. The values of some of these village-specific parameters were available from the datasets collected during the origin and destination field trials, while others were not. Among the latter, we can include features such as the extension of outdoor human defecation and pig roaming areas around village households. Local factors such as characteristics of land cover around households, household density and village population habits can certainly have an influence in determining the exact extension of these areas around each household. To generate the extension of household contamination and pig roaming areas, we used probability distributions, which were not changed from village to village and that were determined from studies conducted in villages in the same region as the simulated villages [22, 41]. Other parameters that can be considered local but for which we used the same values for all simulated villages are human movement rates and pig and pork import rates. We do not have data about the values of these parameters for each simulated village, but forthcoming studies will determine the human movement and pig trade rates in an entire region, which should help update the model accordingly. The group of local parameters for which values were determined during field trials are parameters connected with household location, household human and pig composition and latrine and corral distribution and use. The effect of changing these parameters on the level of transmission is evident in specific villages. For example, in the origin village v515, a relatively high level of transmission among pigs (values of PCP in Fig. 4) corresponds to a low proportion of households owning a latrine (22%) compared to an average latrine ownership proportion of 62% across the origin villages group. On the other hand, village v507 presents a low PCP value presumably attributable to a high proportion of corral use (78% vs. 30% on average in origin villages). For the remaining origin villages, transmission levels are the product of a more complex combination of parameter values.

One of the objectives of this model was to reduce the complexity of data collection processes required to apply the model to new villages. The model does rely on a group of parameters (contextual data) whose values have to be collected for each new simulated village, but it is important to note that these do not depend on time-consuming and expensive screening activities requiring biological material collection and that a census covering all village households is sufficient to collect all the necessary information. We have therefore indeed progressed toward our objective of reducing complexity in data collection needs associated with the model.

Finally, the model described here represents a preliminary step towards the design and implementation of a large-scale ABM transmission to simulate the *T. solium* transmission in an entire region that will include small urban areas, multiple villages, and all human and pig movements and pork trades within the area. For regions not dissimilar to Piura rural areas, the calibrated and transferable CystiAgent parametrization of this study could be exported to all the villages within the region without any further adjustment. The resulting large-scale model will be useful in studying *T. solium* and other cestode parasites for various reasons. First, a large-scale ABM will enable the investigation of regional transmission dynamics after intervention. The resurgence of transmission to pre-intervention levels, even after successful eradication or control campaigns, has been frequently attributed to pathogen flows caused by the movement of people and to pig and pork trades, which bring back the parasite to previously intervened areas. Once an entire region is represented in the model, it will be possible to study sustainable intervention strategies in the long term, examining and controlling for the impact of pathogen flow on transmission resurgence. Second, if economic and health costs and benefits of interventions and diseases are included in the model, it should be possible to study the entire spectrum of economic and spatial feedbacks involved in the establishment of the endemic *T. solium* transmission equilibrium in the region. Therefore, the model will be able capture the socioeconomic, temporal and spatial heterogeneities associated with the complex dynamics of *T. solium* transmission, allowing for the design and study of control end elimination campaigns based on regional, village-specific, geographically targeted and individual-based interventions.

### Supplementary Information

**Additional file 1: Figure S1** Observed pig seroprevalence in households hosting a tapeworm carrier as a function of the deciles of households distance from the village geographical center.

## Data Availability

The program code used for simulations and calibrations is available on a public GitHub repository at https://github.com/oflixs/CystiAgent_04-2023 but the individual-level data used as inputs are only available as de-identified and aggregated, to protect the confidentiality and privacy of re-search subjects.
