# Supplementary material for: Spatial transferability of an agent-based model to simulate Taenia solium control interventions
Source: Parasit Vectors. 2023 Nov 8;16:410. doi: 10.1186/s13071-023-06003-9 (PMC10634186; doi:10.1186/s13071-023-06003-9)
Supplement: Supplementary file 1 — Additional file 1: Figure S1 Observed pig seroprevalence in households hosting a tapeworm carrier as a function of the deciles of households distance from the village geographical center. [file 13071_2023_6003_MOESM1_ESM.docx]

Supplementary material 1

# Changes to the CystiAgent model transmission core

CystiAgent was slightly changed compared to the version presented in [1]. The main changes introduced in CystiAgent concern the introduction of a village border effect on latrine use and the introduction of a new parameter, propDefOut which describes the average proportion of defecations each village agent produces outside the contamination range of its household.

## Border effect

The defecation habits of people residing in rural Peruvian villages can be influenced by various factors such as the availability of sanitation infrastructures, cultural habits, convenience and others. Among these factors, the availability of suitable areas for outdoor defecation can play an important role. Typically, women but also men search for a place not to close to households, with convenient privacy, where the disturbance is minimal. For these reasons, the presence of many households and people in crowded areas may discourage outdoor defecation, as people often prefer a private and secluded location for defecation. This may result in increased use of latrines and other sanitation facilities in the village centers as opposed to the village borders, where suitable open defecation places are more readily available. As consequence, a “border effect” is generated that decrease the adherence to sanitation infrastructures in the households located far from the village geographical center.

To verify the existence of this border effect, we considered the 21 villages of the destination dataset. We selected the group of infected households in which a tapeworm carried was found during the final round of human treatment and stool screening of both origin and destination field trial. We then calculated the seroprevalence of pig owned by households in a range of 250 m around the selected infected households, that was observed during the final round pigs of serology screening. The presence of a tapeworm carried together with a decrease use of sanitation infrastructure should produce an increase in pig seroprevalence in the village border compared to the village center. As it showed in Figure S1, we observed a 29% increase in pig serology around the households contained in the last to deciles of distances from the village geographical center.


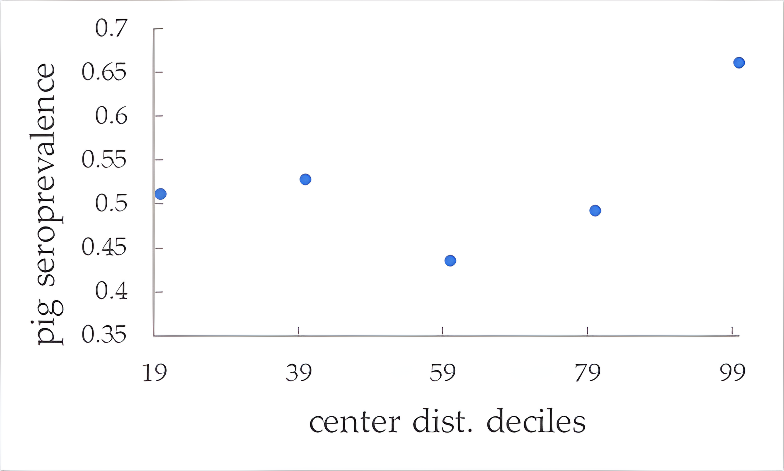


***Figure S1.*** *Observed pig seroprevalence in households hosting a tapeworm carrier as a function of the deciles of households distance from the village geographical center.*

To introduce the border effect in CystiAgent and reproduce the observed increase in the pig seroprevalence of household at the village border, we set the adherence to the use of latrines to 0 in all the simulation for all the member of households lying in the 20% farthest household from the village geographical center.

## Introduction of parameter propDefOut

The newly introduced parameter propDefOut, describes the proportion of villager defecations inside their respective contamination home range. After a series of tests, the value of this parameter was set, in this study to 0.9, meaning that the 10% of defecations of each villager agent are located in areas far from the household and do not contribute to the local spreading of TS among pigs. This parameter was introduced to implicitly take into account two factors. The first factor is the daily human movements, that take the villagers away from home for reasons such as going to work, school or any other nearby destination. The second factor is the existence of areas to which pigs are not allowed, such as agricultural areas that may provide suitable places for outdoor defecation. All defecation inside these areas, even if occur within the household’s contamination radius, which are not accessible to pigs, are considered equivalent to defecations occur far from the household.

**Table S1: variation ranges for calibration parameters that define the prior marginal distributions for the first ABC-SMC stage**

| **Calibration parameter** | **Lower limit** | **Upper limit** |
| --- | --- | --- |
| pHumanCyst | 0.0 | 5 10^-3^ |
| pigProglotInf (cysts/contaminated site) | 10 | 5000 |
| pigEggsInf (cysts/contaminated site) | 0 | 200 |
| seroConvert | 0 | 1000 |
| seroConvertPtoEFact | 0.4 | 1 |

## References

1. Pizzitutti, F.; Bonnet, G.; Gonzales-Gustavson, E.; Gabriël, S.; Pan, W.K.; Pray, I.W.; Gonzalez, A.E.; Garcia, H.H.; O’Neal, S.E.; Cysticercosis Working Group in Peru Non-Local Validated Parametrization of an Agent-Based Model of Local-Scale Taenia Solium Transmission in North-West Peru. *PLoS One* **2022**, *17*, e0275247.
